# Impact of cognitive-behavioral motivation on student engagement

**DOI:** 10.1016/j.heliyon.2022.e09843

**Published:** 2022-07-03

**Authors:** Maninder Singh, P.S. James, Happy Paul, Kartikeya Bolar

**Affiliations:** a Manipal Academy of Higher Education and Faculty Associate, T A Pai Management Institute, India; b Department of HRM & OB, Jagdish Sheth School of Management, Bangalore, India; c Department of HR, OB & Communications, T A Pai Management Institute, Manipal Academy of Higher Education, India; d Department of Operations and Decision Sciences, T A Pai Management Institute, Manipal Academy of Higher Education, India

**Keywords:** Student motivation, Adaptive cognition and behavior, Student engagement, Work engagement, Self-determination

## Abstract

This study explores the relationship between student motivation and student engagement. The study, which is rooted in the self-determination (SDT) and engagement (JD-R) theories, responds to the contemporary call for studying this relationship. A bipartite construct of motivation measures both positive and negative components of motivation and structural equation modeling (SEM) by using data from 693 undergraduate and graduate students. In doing so, the study finds that student motivation is an antecedent of engagement. Adaptive cognition and behavior are positively related to engagement (β = 0.30, β = 0.60); maladaptive cognitions and behavior are negatively related to engagement (β = -0.54). The study advances SDT and JD-R. Implications for educationists and possible interventions to enhance motivation and, consequently, engagement are discussed. The study brings clarity to the student motivation-engagement relationship.

## 1 Introduction

Students’ motivation is a subject of importance; therefore, many studies have tried to address this topic as it leads to desirable outcomes. Of late, scholars working on student motivation suggest that motivation leads to student engagement (Coates, 2005; Furlong and Christenson, 2008; Horstmanshof and Zimitat, 2007). Due to a paucity of literature on the subject (Dincer et al., 2017, 2019; Montenegro, 2017; Noels et al., 2019), this relationship requires more research (Hsieh and Yu, 2022).

This study explores the relationship between student motivation and engagement, which is a growing area of interest among scholars. The context of the study includes university students due to concerns that motivation deteriorates as a person progresses in one's academic career (Wijsman et al., 2016). Dropout rates in college education are substantial. For example, 40% of enrolled students in the United States drop out of higher education (Flynn, 2014). In Europe, the rate is 15%–35% based on the stream of study (Vossensteyn et al., 2015). The rate is 20% in Australia for first-year university students (Shipley and Walker, 2019). Finally, 12.6% out of 38.5 million enrolled students drop out in India (Gulankar, 2020). University education is the gateway to a successful career and life in the modern context; therefore, the motivation and engagement of the university student cohort must be addressed.

The initial stages of the theorization of motivation brought out several theories and concepts, including: the hierarchy of needs (Maslow 1943, 1954); existence, relatedness, and growth (ERG) theory (Alderfer, 1969); Herzberg's two-factor theory (Herzberg et al., 1959; Herzberg, 1965); the need for achievement, power, and affiliation (McClelland, 1958; McClelland and Mac Clelland, 1961); and goal theory (Locke and Latham, 1990; Nicholls, 1984). Intrinsic motivation (what a person inherently wants to do because of internal stimuli) and extrinsic motivation (a response to an external stimulus like praise, rewards, or punishment) are inherent in these theories, even if not explicitly articulated. These theories indicate the multidimensional framework of motivation.

Efforts to consolidate theories led to the self-determination theory (SDT), which encapsulates multiple dimensions (Deci and Ryan, 1985). There is general concurrence on the desirability of using multiple frameworks (Howard et al., 2020; Sheldon et al., 2017). Still, there is a counterview (Chemolli and Gagné, 2014). The core of SDT is autonomy, control, and regulation. SDT can be conceived as a continuum with amotivation (nonresponsiveness to any stimuli) at one end and intrinsic motivation at the other. Extrinsic motivation is in between. Using metanalysis techniques, Howard et al. (2020) elucidated the continuum, suggesting that amotivation can be explained through maladaptive behaviors arising out of factors like low expectancy or value (Wigfield et al., 2017), low self-efficacy (Schunk and DiBenedetto, 2016), and learned helplessness (Abramson et al., 1978).

There are four states of extrinsic motivation between amotivation and intrinsic motivation (Howard et al., 2020). The first, external regulation, results in seeking external awards or avoidance of external punishments. Although it has short-term benefits (Koretz, 2017; Levitt et al., 2016, it is considered a low-quality motivation because it tends to undermine self-determined actions (e.g., Deci et al., 1999). The second, interjected regulation, results in action. It can accommodate several theories like goal theories (Duda, 1989; Nicholls, 1984), self-esteem perspectives (Paradise and Kernis, 2002), and contingent self-esteem (Park and Crocker, 2008) through contingent reward by parents, teachers, or others (Roth et al., 2009). It, too, has a maladaptive connotation because the actions may be driven by guilt or shame avoidance and pride-seeking behavior (Ryan and Deci, 2017). The third, identified regulation, is driven by personal values and beliefs or other variables that lead to action. Unlike intrinsic motivation, these may not be inherently enjoyable; however, they can lead to student outcomes at par with intrinsic motivation (Howard et al., 2020). The fourth form of motivation, integrated regulation, arises when a person has fully integrated different forms of motivation within oneself. Due to the continuum nature of the theory, it is apt to include both adaptive and maladaptive aspects in a study rooted in SDT (Howard et al., 2020).

Though elegant, parsimonious, and generalizable, SDT operationalization is challenged due to its insufficient process perspective. The virtue of intrinsic motivation is well documented; however, it does not fully explain student motivation (Connell and Wellborn, 1991; Howard et al., 2020; Skinner et al., 1990). Perceived control, a new conceptualization consisting of control beliefs, expectations about effective motivation strategies, and the capacity to execute them for the outcome and influence of academic performance (Skinner et al., 1990), was both a reinforcer of SDT and a harbinger of change. Unlike SDT, it can better capture outcome. The self-system model of motivational development (Connell, 1990; Skinner et al., 1990), a theoretical model consisting of competence, autonomy, and relatedness, may overcome SDT deficiencies.

The self-system process model (Connell and Wellborn, 1991) is a more practical conception than SDT; therefore, several studies have adopted this framework (Connell and Wellborn, 1991; Skinner and Belmont, 1993). It conceives motivation as something that depends on context, self, action, and outcome. Its first benefit acknowledges the contingent nature (context) of motivation. In simple terms, a student may be motivated because of one institution's environment; however, the same student may not be motivated due to another environment. The second benefit captures the correlation of “self,” providing a scope to include individual variables like personality, self-efficacy, locus of control, and emotional quotient. These critical variables of self are known to influence motivation. In other words, this framework can help explain why students demonstrate different states of motivation in the same context (for example, the same institution). The third benefit highlights action and outcome orientation. It has the potential to link motivation and engagement when looking at these concepts more holistically.

The engagement construct, which has received considerable attention in management and academics, is considered one of the most critical factors that contribute to learning (Skinner et al., 2009). Engagement is a state; therefore, it can be influenced by contexts, policies, practices, and peer interaction (Sinclair et al., 2003). The study of motivation is concerned with energy, purpose, and sustained action (Skinner et al., 2009). Engagement is focused on vigor, dedication, and absorption (Schaufeli and Bakker, 2004). Therefore, the study of motivation and engagement tends to be intertwined (Skinner et al., 2009).

## 2 Current study

This study acknowledges the benefits of motivation and engagement. Both have an outcome orientation and appear to be intertwined. This study examines whether motivation is an antecedent of engagement. In sum, this study addresses the knowledge gap and calls for further research in the relationship between student motivation and student work engagement proposed by Ryan and Deci (2020).

First, the research establishes the relationship between student motivation and engagement. Second, it enhances the understanding of SDT, the self-system process model, and engagement theories rooted in the job demand-resource (JD-R) model by exploring whether motivation is an antecedent of engagement. The study examines the relationship between adaptive cognitive and behavior, components of motivation, and work engagement of students. Then, it examines the relationship between maladaptive cognition and behavior, as well as components of motivation, on work engagement of students.

### 2.1 Student motivation

Student motivation an important influencing factor on student learning, participation, and academic outcomes, inspiration, self-direction, energization to achieve goals, and effort to learn (Bruinsma, 2004; Ryan and Deci, 2000; Schuetz, 2008; Sternberg, 2005; Zepke et al., 2010). Behavioral and cognitive challenges can result in low motivation, which may lead to poor academic performance (Kahu and Nelson, 2018). While motivation is crucial for academic accomplishment, the quality and quantity of motivation may vary based on time and individual. This will depend on the learning context (Sternberg, 2005).

Faculty uses extrinsic motivation techniques to encourage and stimulate learning through rewards and recognition, free time, punishment, etc (Krause et al., 2006). These initiatives can lead to extra effort by the students (Roebken, 2007). However, the importance and sustainability of higher-order motivation through intrinsic motivation have seldom been disputed (Law et al., 2012; Lee et al., 2010; Vansteenkiste et al., 2004). Studies suggest that intrinsically motivated students report low anxiety. They welcome competition, focus on achievement, and engage more in learning (Wigfield and Wagner, 2005). While types of motivation influence student learning and academic involvement (Saeed and Zyngier, 2012), intrinsic motivation leads to engagement (Wigfield and Wagner, 2005).

Motivation can also be characterized in terms of “boosters” or adaptive cognition and behavior and “guzzlers” or maladaptive cognition and behavior (Martin 2001, 2003, 2007). Boosters are triggered by self-efficacy, planning, task management, mastery orientation, valuing, and persistence. Self-efficacy is “the students' belief and confidence in his/her ability to understand or do well in the course work.” It is the ability to meet challenges and perform one's best. Valuing is “how much students believe what they do and learn at college is useful, important, and relevant to them.” Mastery orientation involves “being focused on understanding, learning, solving problems, and developing skills.” Planning is “how students plan their work and how they keep track of their progress.” Task management refers to “the way students use their time, organize their timetables, and prepare for classes and exams.” A student's persistence is “the capacity of an individual to persist in challenging situations and find ways to do what is required to be done.”

Guzzlers, or maladaptive cognition and behaviors, are negative motivators like anxiety for good academic scores, failure avoidance, uncertainty control, and self-handicapping. Anxiety includes nervousness or worry. Feeling nervous is an “uneasy or sick feeling students get when they think about their college work or academic tasks.” Failure avoidance occurs when “students try to evade doing poorly to avoid disapproval from parents or teachers.” Uncertainty control is the “students’ feeling of uncertainty about academic performance or not having any control.” Self-handicapping is the “involvement of a student in activities other than academic activities” (Martin, 2001, p. 4, 2003 p. 92, 2007 p. 420), leading to adverse academic outcomes. Guzzlers deteriorate motivation.

Conceiving motivation in terms of boosters and guzzlers is in line with SDT. It conceptualizes motivation as a continuum with amotivation at one end and intrinsic motivation at the other. In addition, it provides a simple framework to capture the amount of one's motivation.

Motivation is often conceptualized with a positive driver; however, negative drivers of motivation, including examination anxiety, cannot be ignored (Ouweneel et al., 2011; Salanova et al., 2010; Wiegand and Geller, 2005). Hence, Martin's (2001, 2003) conceptualization of motivation (in terms of booster and guzzlers) is an appropriate framework to study motivation because it captures both its positive and negative drivers.

### 2.2 Student engagement

The student engagement process encompasses the cognitive, physical, behavioral, and emotional involvement of the student (Dismore et al., 2019). In other words, student engagement is a state in which a student puts quality effort into learning and authentic participation into academic activities. According to Trowler (2010, p. 3), student engagement is “the interaction between the time, effort, and other relevant resources invested by student and institution intended to optimize the learning experience.” Through a higher engagement of students, academic institute enhance student learning outcomes, performance, and reputation. Studies have shown a positive relationship between student engagement in academic work and desirable outcomes. For example, engagement is positively related to levels of knowledge acquisition and cognitive development (Pascarella and Terenzini, 1991), effort to learn (Newmann, 1992), self-involvement in learning, pride in learning, mastery of the subject (Kuh, 2009; Saeed and Zyngier, 2012), working with others, transferring knowledge, creative problem solving (Tight, 2020), and academic achievement (Alvarez, 2002; Shah and Cheng, 2019; Tight, 2020; Zyngier, 2008).

Student work engagement is defined as “a positive, fulfilling state comprising vigor, dedication, and absorption in learning” (Siu et al., 2014, p. 980). Vigor is an “individual's ability to invest effort in studies willingly.” Dedication is “a sense of significance, enthusiasm, inspiration, pride, and challenge in academic work.” Absorption is “being fully concentrated and happily engrossed in learning, whereby time passes quickly, and one feels carried away by one's work” (Schaufeli and Bakker, 2010, p. 13). The tripartite construct of engagement (vigor, dedication, and absorption) propounded by Schaufeli et al. (2002) defines work engagement as “a positive, fulfilling work-related state of mind that is characterized by vigor, dedication, and absorption” (Schaufeli and Bakker, 2004, p. 295). This construct of work engagement is rooted in JD-R (Bakker et al., 2004; Demerouti et al., 2001). The basic proposition by Demerouti et al. (2001) is that any activity, including academic activity by a student, places demands on an individual. These include academic workload, time constraints, or contact with others (i.e., faculty, student colleagues, or academic staff). This, in turn, can lead to exhaustion and burnout.

Exhaustion due to an activity is compensated by resources like feedback, faculty support, rewards, control, participation, and a psychologically safe academic environment. If the latter (resources) are inadequate, the result is disengagement.

Bakker et al. (2004) clarified that there are two independent processes in the JD-R model. The first is an energy-driven process (job demands-burnout negative performance). The second is a motivation-driven process (academic resources-engagement positive performance). Studies in student engagement suggest that one should also consider the negative side effects of engagement, including exhaustion due to activities because burnout leads to low engagement (Salmela-Aro et al., 2016). The burnout-engagement relationship is mediated by intrinsic motivation (Cho et al., 2022). JD-R is considered an appropriate model to study this relationship (Jagodics and Szabó, 2022).

### 2.3 Relation between student motivation and student engagement

Student motivation and engagement in learning are critical factors for academic success (Hufton et al., 2002; Woolfolk and Margetts, 2012). Studies explain that even students with high self-efficacy have difficulty comprehending unless they are actively engaged in learning (Dörnyei, 2000; Lin, 2012). Depending on emotional and cognitive factors, students may be highly engaged or disengaged (Bryson and Hand, 2007). In addition, student outcomes are influenced by student motivation and engagement (Frey et al., 2009).

From the discussion, it emerges that both motivation and engagement have a salutary effect on several performance-facilitating factors (or they may be intertwined). However, the relationship between motivation and engagement remains elusive. Keeping this in view, Pintrich (2003) and Ford and Smith (2009) recommended combining research on motivation and engagement. Skinner et al. (2016) suggested that such research could help in creating better interventions. According to scholars, engagement and students’ active, energetic, passionate, and attentive participation in academic work are the results of motivation (Reeve, 2012; Skinner et al., 2009).

Skinner et al. (2009) explained the relationship between student motivation and student engagement as rooted in SDT and the self-system motivational framework (Connell, 1990; Deci and Ryan, 1985; Skinner et al., 1990). Student motivation and student engagement are influenced by a student's experiences, self-perception, and support of teachers and peers. These factors relate to a student's academic objectives, motivation, values, and perceived self-efficacy, resulting in the student's engagement or disengagement (Patrick et al., 2007). Peer support, interactions with the teacher, and a positive learning environment promote student motivation through positive social experiences and classroom behaviors. As a result, student engagement and academic performance increase (Connell et al., 1994; Wentzel, 2003).

It emerges that existing scholarship has emphasized the relationship between student motivation and engagement in learning outcomes (Anderman and Kaplan, 2008; Connell et al., 1994; Wentzel, 2003). However, the relationship is inconclusive. While scholars agree with the intermingling nature of motivation and engagement, Luthans et al. (2007) suggested that the highest levels of engagement are shown by those who feel motivated, have a sense of self-worth, are hopeful, and show enthusiasm about their future, which points to the direction that motivation is an antecedent of engagement. Hence, this study hypothesizes that:

H1 *Adaptive cognitive and behavioral motivation show a statistically significant positive correlation with the work engagement of students.*

H2 *Maladaptive cognitive and behavioral motivation show a negative correlation with the work engagement of students.*

## 3 Method

### 3.1 Sample and data collection

After seeking ethical approval from the T A Pai Management Institute Research Ethics Committee, the questionnaire was administered to the participants who had intimated about the purpose of the study and the voluntary nature of the survey. Data was collected online using a structured questionnaire. Convenience sampling was used for collecting the responses. For the cross-sectional survey, 3,586 online Google Forms questionnaires were e-mailed to students, with 693 useable responses received. Participants included students from different states who were pursuing undergraduate and graduate studies in various disciplines. Table 1 summarizes the demographic profile of respondents.

**Table 1:** Demographic variable sample composition (n = 693).

Category	Percentage (%)	Frequency
**Gender**		
Female	39%	268
Male	61%	425
**Age Group**		
20–25 years	77%	534
25–30 years	23%	159
**Education**		
Graduates	67%	465
Undergraduates	33%	228
**Family Income**		
Below INR 500,000	15%	104
INR 500,001 to 1 million	38%	263
1.1 to 1.5 million	22%	152
1.6 to 2 million	13%	91
Above 2 million	12%	83

### 3.2 Measurement instrument

#### 3.2.1 Student motivation

Student motivation was measured using a 10-item student motivation scale (SMS) developed by Martin (2001). The adaptive cognitions and behavioral motivation items included self-efficacy, valuing, mastery orientation, planning, persistence, and task management. Maladaptive cognition and behavioral motivation items were related to anxiety, failure avoidance, uncertain control, and self-handicapping. Responses were collected on a five-point Likert scale anchored from “strongly disagree” to “strongly agree.” The responses reported adequate reliability (α = 0.7, CR = 0.7) and validity (AVE = 0.5).

#### 3.2.2 Student engagement

Student engagement was measured using nine items of the Utrecht Work Engagement Scale-Student (UWES-S) survey developed by Schaufeli et al. (2002). The three components of engagement (i.e., vigor, dedication, and absorption) were measured using a five-point Likert scale. The responses reported adequate reliability (α = 0.8, CR = 0.9) and validity (AVE = 0.5). Table 2 shows a summary of items operationalizing all the constructs.

**Table 2:** Summary of variables and respective questionnaire items.

Item Code	Item Description	Construct
AC1	I believe I can do well in my coursework by working hard.	Adaptive Cognition (AC), Adaptive Behaviors (AB), Maladaptive Cognitions (MC), and Maladaptive Behaviors (MB)
AC2	Learning at college is important to me.	
AC3	I am very pleased with myself when I fully understand what I'm taught in class.	
AB1	Before I start an assignment, I plan how I am going to do it.	
AB2	I usually study in places where I can concentrate.	
AB3	If I don't understand a subject, I review it until I understand.	
MC1	I worry a lot when exams and assignments are coming up.	
MC2	I am often unsure how to avoid doing poorly in my coursework.	
MC3	I usually work at college because I want to please my family.	
MB1	I sometimes don't study very hard before exams so I have an excuse if I do poorly.	
V1	When I study, I feel mentally strong.	Student Engagement (SE)
V2	When I study, I feel like I am bursting with energy.	
V3	When I study, I feel strong and vigorous.	
D1	I find my studies to be full of meaning and purpose.	
D2	I am inspired by my studies.	
D3	I am enthusiastic about my studies.	
A1	When I am studying, I forget everything around me.	
A2	I am happy when I'm studying intensively.	
A3	I can get carried away by my studies.	

### 3.3 Data analysis and interpretation

A three-step data analysis was conducted. First, the exploratory factor analysis was performed to determine the underlying dimensions of cognitive-behavioral motivation and student engagement. This was followed by a confirmatory factor analysis (CFA) to verify the factor structure of the constructs. Cronbach's alpha, average variance extracted (AVE), and composite reliability (CR) were calculated to fulfill the reliability and validity criteria. Structural equation modeling (SEM) was used for hypothesis testing via the AMOS (22.0) software package. The model fit indices like comparative fit index (CFI), the goodness of fit index (GFI), normed fit index (NFI), root mean square error of approximation (RMSEA), and chi-square by a degree of freedom were used to indicate model fit with the data.

### 3.4 Descriptive statistics

The statistical test checked the normal distribution of the collected data. All variables were within the normal range of kurtosis and skewness indices (Kline, 2015); therefore, all items were used in subsequent analyses. The descriptive statistics of the measurement items are shown in Table 3.

**Table 3:** Descriptive statistics.

Items	Mean	Median	Std. Deviation	Skewness	Kurtosis
I believe I can do well in my coursework by working hard.	4.3	4.0	0.8	-1.1	1.2
Learning at college is important to me.	4.2	4.0	0.8	-0.8	0.0
I am very pleased with myself when I fully understand what I'm taught in class.	4.2	4.0	0.8	-0.7	-0.1
Before I start an assignment, I plan how I am going to do it.	4.0	4.0	0.9	-0.7	0.0
I usually study in places where I can concentrate.	4.1	4.0	0.9	-0.8	0.0
If I don't understand a subject, I review it until I understand.	3.7	4.0	1.0	-0.4	-0.6
I worry a lot when exams and assignments are coming up.	2.5	2.0	1.2	0.4	-0.7
I am often unsure how to avoid doing poorly in my coursework.	2.6	2.0	1.2	0.3	-0.9
I usually work at college because I want to please my family.	2.8	3.0	1.3	0.3	-1.1
I sometimes don't study very hard before exams so I have an excuse if I do poorly.	2.9	3.0	1.3	0.1	-1.1
When I study, I feel mentally strong.	3.9	4.0	0.9	-0.4	-0.5
When I study, I feel like I am bursting with energy.	3.5	4.0	1.1	-0.3	-0.7
When I study, I feel strong and vigorous.	3.6	4.0	1.0	-0.4	-0.6
I find my studies to be full of meaning and purpose.	3.3	3.0	1.2	-0.1	-0.9
I am inspired by my studies.	3.8	4.0	1.0	-0.5	-0.3
I am enthusiastic about my studies.	3.8	4.0	1.0	-0.4	-0.4
When I am studying, I forget everything around me.	3.6	4.0	1.1	-0.5	-0.5
I am happy when I'm studying intensively.	3.8	4.0	1.0	-0.7	-0.1
I can get carried away by my studies.	3.5	4.0	1.2	-0.4	-0.7

### 3.5 Exploratory factor analysis

The suitability of the data for factor analysis was assessed before performing the factor analysis. The Kaiser-Meyer-Olkin measure of sampling adequacy (MSA) was 0.843. This value exceeded the recommended value of 0.6. Further, Bartlett's test of sphericity reached statistical significance, which supported the correlation matrix's factorability. EFA was performed on the 19 items of the measurement scales using SPSS 22. A principal component analysis with varimax rotation was used to identify the underlying dimensions related to student motivation and student engagement. The criteria used for factor extraction were two-fold. The eigenvalue should be greater than one and the factor structure should be meaningful and conceptually correct (Pett et al., 2003). Retained factor loadings were greater than 0.50 for further analysis. In total, 16 items loaded appropriately on the four factors: (1) student engagement; (2) adaptive cognition; (3) adaptive behavior; and (4) maladaptive cognition and behavior.

These four factors accounted for 51% of the total variance explained. Three items from the student work engagement were removed because they did not load on any factors. Cronbach's coefficient alpha values of subscales were acceptable (0.7 or above); hence, the factors were reliable (Hair et al., 2014). This indicated a reliable measurement instrument. Table 4 shows the factor structure, factor loading, and reliability measure of Cronbach's coefficient alpha.

**Table 4:** Exploratory factor analysis and Cronbach's alpha.

Variable	Construct	Items	Loadings	Cronbach's Alpha
Student Engagement	Work Engagement	Vigor	0.7	0.8
		Vigor	0.7	
		Dedication	0.6	
		Dedication	0.7	
		Absorption	0.6	
		Absorption	0.6	
Student Motivation	Adaptive Cognitions	Self-efficacy	0.7	0.7
		Valuing	0.6	
		Mastery orientation	0.7	
	Adaptive Behavior	Planning	0.5	0.7
		Task management	0.6	
		Persistence	0.6	
	Maladaptive Cognition and Behavior	Anxiety	0.7	0.8
		Failure avoidance	0.7	
		Uncertain control	0.7	
		Self-handicapping	0.6	

Extraction Method: Principal Component Analysis. Rotation Method: Varimax with Kaiser Normalization.

### 3.6 CFA

CFA was carried out to examine the reliability and validity of the proposed constructs using AMOS 22.0 (Hair et al., 1998). The model fit indices were considered to assess the GFI of the proposed measurement model. The details of the model fit indices of the measurement model. The recommended values are presented in Table 5. All the model fit indices for the measurement model were acceptable with a chi-square by a degree of freedom value 3.84 (McIver and Carmines, 1981). This result suggests that the data collected from the respondents is aligned with the items reflected in the constructs.

**Table 5:** Model fit indices for the measurement model.

Model Fit Indices	Recommended Value	Measurement Model
Chi-square to the degree of freedom ratio (CMIN/df)	Between 1 and 5	3.84
Goodness of fit index (GFI)	0.90 or above	0.93
Adjusted goodness of fit index (AGFI)	0.80 or above	0.91
Normed fit index (NFI)	0.80 or above	0.86
Comparative fit index (CFI)	0.80 or above	0.89
Parsimony normed fit index (PNFI)	0.60 or above	0.69
Parsimony comparative fit index (PCFI)	0.60 or above	0.72
Root mean square of error approximate (RMSEA)	0.070 or below	0.064

Next, the validity of the constructs was assessed. Churchill (1979) suggested that convergent and discriminant validities should be examined for construct validity. Therefore, the study assessed convergent validity by examining CR and AVE from the four constructs (Hair et al., 1998). The CR of all the factors was equal to or above the recommended value of 0.70 (Bagozzi and Yi, 1988). The AVE values were equal to or above 0.50, supporting the convergent validity. Table 6 indicates the AVE and CR values of all four constructs.

**Table 6:** CR and AVE.

Variable	Factor	Measurement Items	Standardized Estimates	AVE	CR
Student Engagement	Student Work Engagement	Vigor	0.7∗	0.5	0.9
		Vigor	0.8∗		
		Dedication	0.7∗		
		Dedication	0.7∗		
		Absorption	0.8∗		
		Absorption	0.7∗		
Student Motivation	Adaptive Cognitions	Self-efficacy	0.7∗	0.5	0.7
		Valuing	0.6∗		
		Mastery orientation	0.7∗		
	Adaptive Behavior	Planning	0.7∗	0.5	0.7
		Task management	0.7∗		
		Persistence	0.7∗		
	Maladaptive Cognition and Behavior	Anxiety	0.7∗	0.5	0.8
		Failure avoidance	0.7∗		
		Uncertain control	0.7∗		
		Self-handicapping	0.7∗		

Note: ∗Implies that the estimate values are statistically significant at p < 0.001.

For the discriminant validity, as shown in Table 7, the value of AVE of each construct was greater than the square of the inter-construct correlations. This, thus, satisfies the discriminant validity criteria (Fornell and Larcker, 1981).

**Table 7:** Discriminant validity.

	Adaptive Cognitions	Adaptive Behavior	Maladaptive Cognition and Behavior	Student Engagement
Adaptive Cognitions	**0.51**	0.38	0.01	0.16
Adaptive Behavior	0.38	**0.52**	0.10	0.35
Maladaptive Cognition and Behavior	0.01	0.10	**0.50**	0.34
Student Engagement	0.16	0.35	0.34	**0.53**

Note. The values in bold are the AVE.

### 3.7 Hypothesis testing

**Figure 1:**
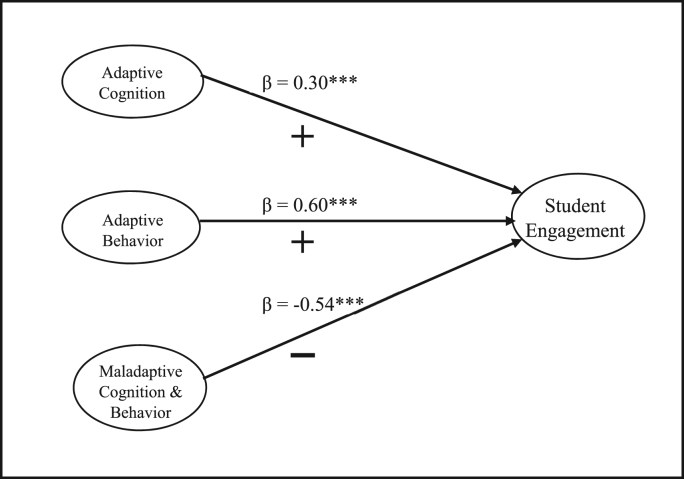
Hypothesis Testing- Relation between Cognitive and Behavioral Dimensions of Motivation and Student Work Engagement. Note. ∗∗∗p = 0.001.

The relation between student motivation and student engagement was statistically tested using SEM (AMOS 22.0). The association between adaptive cognitions, adaptive behaviors, and maladaptive cognitions and behavior was tested with student work engagement. Results are shown in Figure 1. Results indicate an adequate model fit with the data. Model fit indices like chi-square/degrees of freedom = 4.5 (<5); GFI = 0.91; NFI = 0.86; CFI = 0.85; RMSEA = 0.07; IFI = 0.85; and PNFI = 0.68 further validated the hypothesized association between constructs.

The result demonstrates a statistically significant positive relation between adaptive cognitive motivation (self-efficacy, valuing, and mastery) and student work engagement (β = 0.30, p = 0.001). There is a statistically significant positive relation between adaptive behavioral motivation (planning, task management, and persistence) and student work engagement (β = 0.60, p = 0.001). Maladaptive cognitions and behavior (anxiety, failure avoidance, uncertain control, and self-handicapping) are negatively associated with student work engagement (β = -0.54, p = 0.001). Thus, both H1 and H2 are found to be statistically significant.

## 4 Discussion

This study examined the relationship between student motivation and student work engagement in the context of university education. To be holistic, the study used both positive (booster) and negative (guzzler) factors of motivation (Martin, 2001, 2003, 2007), as well as a tripartite construct of engagement that consisted of vigor, dedication, and absorption (Schaufeli et al., 2002). The study hypothesized that adaptive cognition and behavior components (boosters) of motivation would show a statistically significant positive correlation with student work engagement. In addition, it posited that maladaptive cognition and behavior (guzzler) would show a statistically significant negative correlation with student engagement.

This study found support for both hypotheses. Boosters (adaptive cognition and behavior) show a statistically significant positive relationship with student work engagement. In contrast, guzzlers (maladaptive cognition and behavior) show a statistically negative relationship with student work engagement. In other words, positive motivation factors lead to work engagement and negative motivation factors lead to disengaged students.

The findings of this study highlight that boosters and guzzlers have a different association with students’ work engagement. The conceptualization of motivation is often embedded in positive factors, which, in turn, lead to motivation (Ouweneel et al., 2011; Salanova et al., 2010; Wiegand and Geller, 2005). Still, the existence of negative motivators (i.e., examination anxiety and a feeling of helplessness) cannot be ignored. The results suggest that studies on motivation should consider both aspects: factors/dimensions that support motivation and those that work against motivation.

The result of the study suggests that the advancement of an individual's motivation for achievement could be achieved by increasing the boosters and controlling the guzzlers. Thus, the study supports the bipartite conceptualization of motivation (Martin, 2001, 2003, 2007). The result aligns with SDT, which conceptualizes motivation on a continuum from amotivation to intrinsic motivation (Howard et al., 2020). It, thus, reinforces SDT.

An important finding of this study is that motivation is an antecedent of engagement. This helps to clarify the relationship between student motivation and student work engagement. The result can be considered robust because both positive and negative factors of motivation (boosters and guzzlers) show the antecedence. Boosters are positively correlated; guzzlers are negatively correlated. Thus, the study suggests that motivation is a critical factor on work engagement. These findings are in line with the work of Luthans and Youssef-Morgan (2017), who suggested that motivation is the most important predictor of engagement.

The findings of this study support the relationship between motivation and engagement in the educational context. They also reinforce the engagement concept rooted in JD-R. Thus, the study extends the application of work engagement to the education field, which is in line with earlier studies related to motivation and engagement (Bryson and Hand, 2007; Hufton et al., 2002). A unique piece to this study is that it concurrently tests SDT and JD-R (Deci and Ryan, 1985; Demerouti et al., 2001). It also validates the bipartite construct of motivation (Martin, 2001, 2003) and tripartite construct of engagement (Schaufeli et al., 2002).

## 5 Implications

Though studies suggest that motivation and engagement are intertwined (Skinner et al., 2016), this study found that student motivation is the antecedent of student work engagement in the context of university students. Another implication is that positive and negative motivation factors showed a different relationship with engagement (hence, the need to consider both positive and negative factors in studies related to motivation).

Educators could note the strong positive correlation of 0.6 with adaptive behavior and a near-equal negative correlation of maladaptive behavior (0.56) with engagement. This suggests that the route to engagement is not only through modification of positive motivation generating behavior but also through controlling the negative motivation generating behavior. Educators can use motivation as a tool to create student work engagement through customized intervention. Using the bipartite construct of motivation, educators can cluster students into groups using a simple 2 × 2 matrix as follows: (1) high boosters and low guzzlers; (2) low boosters and low guzzlers; (3) low boosters and high guzzlers; and (4) high boosters and high guzzlers.

**Figure 2:**
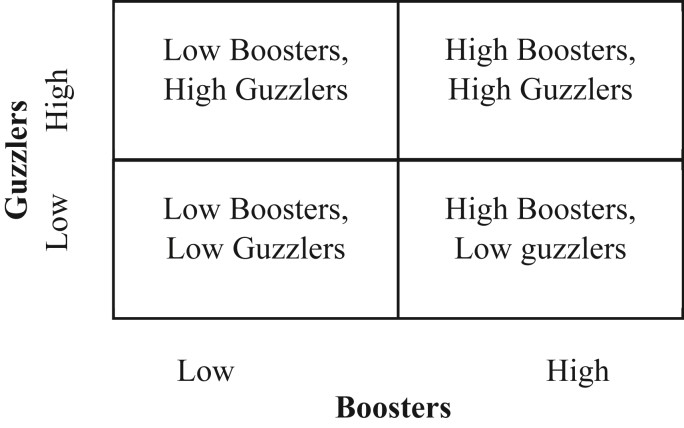
A model for clustering students for motivational interventions.

No intervention is required for the first category (see Figure 2). This category would tend to be engaged. Positive reinforcement would help the second category. For example, faculty can use tools like self-assurance, challenging tasks with mentoring and feedback, goal setting, and work planning to enhance motivation. The tools recommended for the second category may also be applicable to the third category. However, the third group may need specialist intervention if the guzzler effect is strong or needs to address a specific issue like anxiety. The fourth group is high potential if the guzzlers are addressed through positive talk and, if required, specialist intervention.

Engagement can be enhanced through institutional-level interventions like creative course content, flexible and hybrid learning programs, or facility support. However, considering the antecedent nature of motivation, institutions may be surprised that many of their interventions may not yield the expected results. This scenario could change if motivation, the antecedent of engagement, is addressed as discussed.

Strategies and tools are essential to motivate students. These include clear messaging to students or highlighting learning objectives, reasons for learning, expectations from students, the structure of coursework, and the learning and assessment process. Student seminars and workshops on academic planning, subject-specific guidance, developing a focused approach, setting term or semester goals, breaking lengthy projects and assignments into smaller components, self-assessment, and improvement methods can build a positive, success-driven academic culture for students. These efforts will lead to motivation and engagement. While these are well-known tools, they are often used as a generic tool with salutary effect on some but little or no effect on others. Clustering would make it possible to customize motivation tools to enhance engagement.

Clustering is an institutional-level intervention. However, the study can create individual motivation strategies for enhancing student work engagement. In addition, it can help apply resources to better educate a student. For example, task-based feedback could provide clear directions for students to improve in their academic engagement (de Borba et al., 2020). For students with high fear of failure, faculty could reposition success as personal growth and development rather than the outperformance of others. Teachers can shape a student's attitude to see mistakes and failure as lessons for future success. Institutions could create student support systems like e-counseling and mentor-mentee programs to help students understand the gap between their efforts, academic performance, and areas of improvement (Tani et al., 2021). All these initiatives would lead to higher student work engagement.

## 6 Conclusion

The relationship between student motivation and engagement may be intertwined (Skinner et al., 2016). However, the study shows that student motivation is an antecedent to student work engagement. The positive and negative behaviors related to motivation influence have different impacts on engagement; therefore, one cannot ignore the guzzlers when creating interventions. Measuring motivation using the bipartite construct (Martin, 2001, 2003) or similar models simultaneously can create clusters of students who can design a customized intervention to achieve student work engagement. The relationship between student motivation and student work engagement demands serious studies if university student performance is to be enhanced.

### 6.1 Limitations

This study explains the value of understanding both adaptive and maladaptive cognition and behavior. It also explores ways to address these issues to enhance engagement. However, this study has its limitations. First, it does not explain important individual differences between the students. For example, how do personality traits affect the boosters and guzzlers? Big five personality factors like conscientiousness or neuroticism influence student engagement (Qureshi et al., 2016); therefore, understanding the moderating effect of such factors should be included in future studies. Second, the study does not consider the emotional quotient of the students, which is likely to moderate the effect of guzzlers (Bautista et al., 2018). Third, it would be insightful if future studies embarked on longitudinal studies on the impact of interventional strategies using random control treatment (RCT) experiments related to student engagement.

## Declarations

### Author contribution statement

Maninder Singh:Conceived and designed the experiments; Performed the experiments; Analyzed and interpreted the data; Contributed reagents, materials, analysis tools or data; Wrote the paper.

P.S. James:Conceived and designed the experiments; Performed the experiments; Wrote the paper.

Happy Paul:Conceived and designed the experiments; Analyzed and interpreted the data; Contributed reagents, materials, analysis tools or data.

Kartikeya Bolar:Analyzed and interpreted the data; Contributed reagents, materials, analysis tools or data.

### Funding statement

This research did not receive any specific grant from funding agencies in the public, commercial, or not-for-profit sectors.

### Data availability statement

The authors do not have permission to share data.

### Declaration of interests statement

The authors declare no conflict of interest.

### Additional information

No additional information is available for this paper.
